# Integrative analysis of chemical properties and functions of drugs for adverse drug reaction prediction based on multi-label deep neural network

**DOI:** 10.1515/jib-2022-0007

**Published:** 2022-05-19

**Authors:** Pranab Das, Yogita, Vipin Pal

**Affiliations:** National Institute of Technology Meghalaya, Shillong, India

**Keywords:** adverse drug reactions, chemical properties of drugs, deep neural network, drug functions

## Abstract

The prediction of adverse drug reactions (ADR) is an important step of drug discovery and design process. Different drug properties have been employed for ADR prediction but the prediction capability of drug properties and drug functions in integrated manner is yet to be explored. In the present work, a multi-label deep neural network and MLSMOTE based methodology has been proposed for ADR prediction. The proposed methodology has been applied on SMILES Strings data of drugs, 17 molecular descriptors data of drugs and drug functions data individually and in integrated manner for ADR prediction. The experimental results shows that the SMILES Strings + drug functions has outperformed other types of data with regards to ADR prediction capability.

## 1 Introduction

Drug discovery and design is a complex process and is the backbone of the pharmaceutical industry. It involves lots of efforts with regard to time and cost. Many of the drugs get failed and are withdrawn from the market because of their adverse reactions. Adverse drug reactions (ADR) are defined as the undesired affects of a drug which occur even on consuming the prescribed dose of a drug [1]. The timely prediction of ADR based on different drug properties can be very helpful to save the time and other resources. Machine learning (ML) can play a vital role in ADR prediction [2–4]. Deep learning is a sub-branch of ML which deals with the deep neural networks (DNN) having multiple hidden layers. DNN learns the data abstraction in form of relevant features automatically from the given data which enhances its aptness for being used in ADR prediction.

The problem of ADR prediction is multi-label in nature as more than one ADRs can be associated with a single drug. Further, it faces the challenge of small data size where some of the drugs (data samples) across different ADRs (labels) are under represented. The objective of the present work is to analyse the prediction capability of two chemical drug properties namely – 17 molecule descriptors and SMILES Strings, and drug functions individually and in integrative manner towards ADR prediction. The underlying principle towards ADR inferring capability of SMILES Strings is that the drugs with similar chemical structure induce similar drug target binding profiles (control similar biological pathways) and hence leads to similar ADRs. Further, the drugs with similar chemical structure can be deployed for different drug functions and if so then drugs even having same chemical structure being used for different drug functions can have different ADRs. Hence the SMILES Strings and Drug Functions ADR prediction capability has been analysed individually and in integrated fashion. For this purpose, a multi-label deep neural network (MLDNN) based methodology has been presented in this work. It employs Multilabel Synthetic Minority Over-sampling Technique (MLSMOTE) technique [5] to address the issue of data under representation by data augmentation. Though there are other data augmentations techniques such as over sampling, SMOTE etc. but these techniques are inapt for multi-label datasets whereas MLSMOTE is designed specifically for multi-label datasets which works by incorporate the multiple labels while generating synthetic samples hence it has been considered appropriate for the data under study. The proposed methodology has been applied and analysed on seven drug properties datasets extracted from PubChem database [6] and SIDER databases [7] and integrated by mapping on drugs in terms of precision, recall, F1-score, ROC-AUC and Hamming Loss (HL). The main contributions of the present work are:

– A multi-label deep neural network and MLSMOTE based methodology to predict ADRs.
– The analysis of ADR prediction capability of 17 molecular descriptors of drugs, SMILES Strings of drugs and drug functions individually and in integrated manner has been presented.

Rest of this paper is organised as follows: Section 2 details the related work and Section 3 describes the problem statement addressed in the current work. Section 4 explains the proposed methodology to predict ADRs. Section 5 discusses the experimental results. Finally, Section 6 concludes the work.

## 2 Related work

This section provides a brief survey of the works related to ADR prediction using different machine learning and deep learning techniques and highlights the research scope.

In Jamal et al. [8], the author used support vector machine to predict neurological adverse drug reactions from phenotypes, chemical, and biological properties. The author applied the proposed methodology on a single-level dataset, two-level dataset, and three-level dataset and found that the model’s performance is better when phenotypic and chemical properties are combined and compared to other combination datasets. Liu et al. [9] proposed a large-scale prediction of adverse drug reactions using phenotype, biological and chemical structure using support vector machine, random forest, *k* nearest neighbor, naïve Bayes, and logistic regression. The author performed the proposed methodology on single, two, and three-level datasets. Further, the author obtained that their proposed methodology achieved better phenotypic properties when the support vector machine is used.

In Jamal et al. [10], the author predicts adverse drug reactions for cardiovascular drugs using biological information, chemical information, therapeutic indication, and their two-level and three-level combinations. The author used random forest, support vector machine, and Sequential minimization optimization and addressed class imbalance issue using the SMOTE technique. Lee et al. [11] proposed a three-interval method to predict adverse drug reactions by integrating chemical and biological properties of drugs, and the proposed method achieved better results compared to naive Bayes, *k* nearest neighbor, and random forest classifies. In [12], the author proposed a hybrid clustering-based methodology for determining quantitative relationships between adverse drug reactions and patient attributes. Das et al. [13] proposed a multi-label machine learning methodology by utilizing drug functions to predict adverse drug reactions with a class imbalance handling technique named MLSMOTE.

Wang et al. [14] proposed a deep neural network model for predicting adverse drug reactions by combining 17 molecules of chemical and physical computed properties, biological properties, and biomedical research article information. In Uner et al. [15], the author proposed a deep learning framework for predicting adverse drug reactions from gene expression, gene ontology, chemical structure, and META information. The author evaluated the proposed deep neural network model (multi-layer perceptron, residual multi-layer perceptron, multi-modal neural network, and multi-task neural network) at each dataset. After that, they combined two-level and three-level drug properties and obtained that the combination of META data, gene expression, and chemical structure achieved better results than others.

Overall, it can be said that different types of drug properties have been considered for ADR prediction but the integration of drug properties and drug function for ADR prediction is yet to be explored. This observation frames the scope for the present work.

## 3 Problem statement

**Figure 1:**
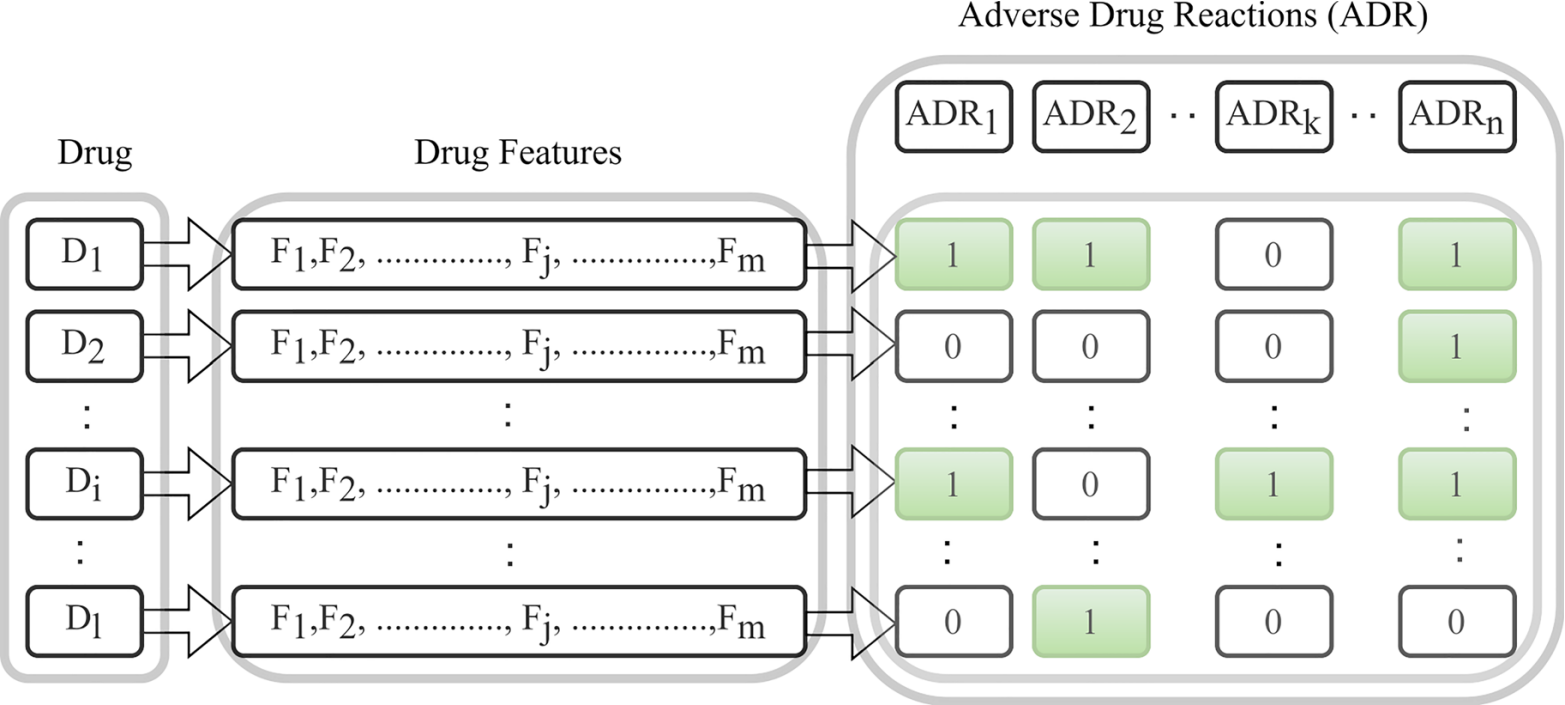
Problem statement.

The problem to predict ADRs based one the integration of drug properties and Drug Functions (DF) is stated as follows:

Let *D* = {*D*_1_, *D*_2_, …, *D*_*p*_, …, *D*_*l*_} be the set of drugs, *F* = {*F*_1_, *F*_2_, …, *F*_*q*_, …, *F*_*m*_} be the set of drug features where each features set represent either a drug property or drug functions or features resulted on integrating one drug property with another drug property or with drug functions or multiple drug property with drug functions. Let ADR = {ADR_1_, ADR_2_, …, ADR_*r*_, …, ADR_*n*_} be the set of ADRs. A drug (*D*_*p*_) can have multiple ADRs. Therefore, the prediction of ADRs for a drug is a multi-label classification problem. Here, *F* and ADR are represented by 1 and 0, where 1 indicates the presence and 0 indicates the absence of *F* and ADR for a specific drug. The diagrammatic view of the problem statement has been shown in Figure 1.

## 4 Proposed methodology

**Figure 2:**
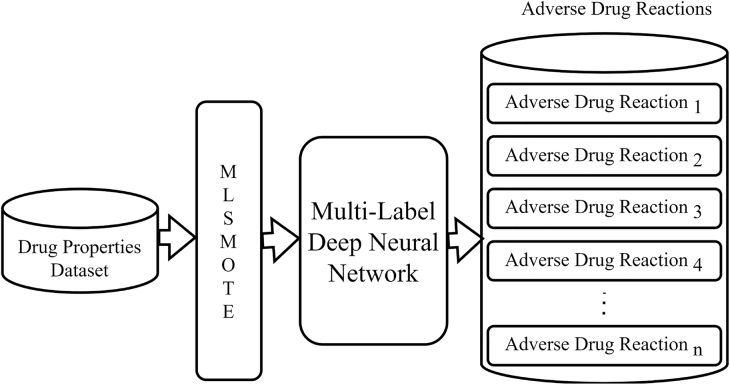
Architecture of the proposed methodology for ADR prediction.

The architecture of the proposed methodology for ADR prediction has been given in Figure 2. It takes 7 drug properties datasets as input and then perform data augmentation using MLSMOTE technique. After that MLDNN models are trained to predict ADRs based on augmented data. The ADRs are provided as output. The details of datasets, MLSMOTE and MLDNN have been given in subsequent sections.

### 4.1 Dataset

**Figure 3:**
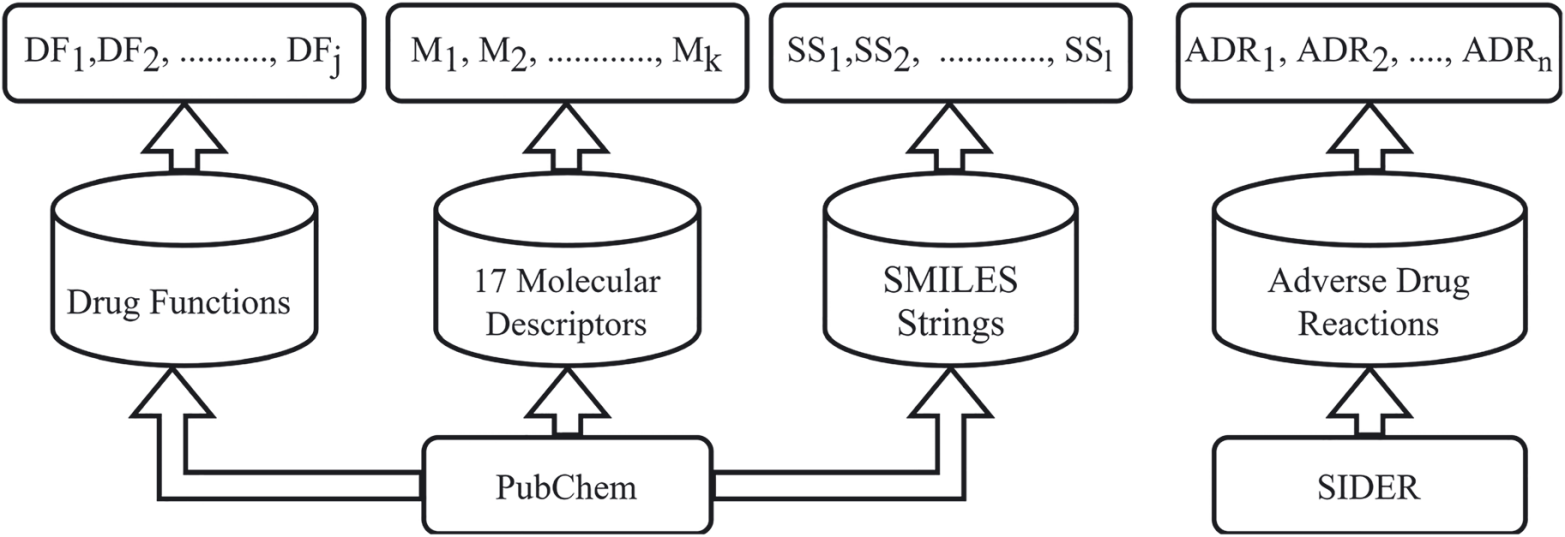
Extraction of data related to drug properties, drug functions and ADRs.

This section initially gives the details of 17 molecular drug descriptors data, SMILES Strings data, Drug functions data and ADR data and then discusses integration of these to prepare 7 datasets for validation of the proposed methodology. The pictorial representation of which has been given in Figure 3.

– **17 Molecular Drug Descriptors Data:** The data related to 17 molecular drug descriptors has been extracted from the PubChem database [6]. It consists of 1430 drugs (data samples) and 17 drug features viz. exact mass, hydrogen bond donor count, covalently-bonded unit count, molecular weight, rotatable bond count, undefined bond stereocenter count, complexity, defined bond stereocenter count, monoisotopic mass, topological polar surface area, isotope atom count, formal charge, heavy atom count, defined atom stereocenter count, hydrogen bond acceptor count, and XlogP3 denoted as M_1_ … M_*k*_ in Figure 3.
– **SMILES Strings Data:** It gives the one dimensional representation of drug molecules in form of predefined sub structures. 1430 drug samples have been extracted from PubChem database [6]. The Morgan Fingerprint of 167 bit length has been used as SMILES embedding for converting ID representation to numeric strings. Morgan Fingerprint is a string of 0’s and 1’s, where 0 indicates the absence of a chemical substructure and 1 indicates the presence of a chemical substructure in a drug. Each bit signify an attribute of drugs indicated as SS_1_ … SS_*l*_ in Figure 3.
– **Drug Functions Data:** These data has been extracted from PubChem database [6] and comprises 670 drug samples. Each sample is described with regard to 12 features (drug function) namely anti-infective, anti-inflammatory, antineoplastic, cardiovascular, central nervous system agents, dermatologic agents, gastrointestinal, hematologic agent, lipid regulating, reproductive control, respiratory system agents, urological denoted as DF_1_ … … DF_*j*_ in Figure 3. The occurrence of a drug function for a given drug is indicated as ‘1’ and its non-occurrence is indicated as ‘0’.
– **Adverse Drug Reactions Data:** The data related to adverse drug reactions has been extracted from SIDER database [7]. It comprises 1430 drug samples where corresponding to each sample information with regard to occurrence and non-occurrence of 6123 ADRs has been provided which are indicated as ADR_1_ … ADR_*N*_ in Figure 3.

For preparing the datasets for the validation of the proposed methodology, firstly three single level datasets namely SMILES Strings (SS) dataset, 17 Molecular Descriptors (MD) dataset and Drug Functions (DF) dataset are created by mapping SMILES strings of drugs, 17 molecular descriptors of drugs and drug functions with ADR data on drug id. Secondly, two-level datasets are created by integrating SMILES strings of drugs with 17 molecular descriptors of drugs, SMILES strings of drugs with drug functions and 17 molecular descriptors of drugs with drug functions and then mapping integrated data with ADRs data on drug id, it resulted three datasets viz. SS + MD, SS + DF and MD + DF. Further, a three level datasets is generated by integrating SMILES strings, 17 molecular descriptors and drug functions all together and then mapping with ADRs data on drug id, it is titled as SS + MD + DF dataset. The statistics of all 7 datasets have been given in Table 1.

**Table 1:** Dataset statistics.

Dataset	No. of data samples	No. of features
SMILES strings (SS)	1430	167
17 molecular descriptors (MD)	1430	17
Drug functions (DF)	670	12
SMILES strings + 17 molecular descriptors (SS + MD)	1430	184
SMILES strings + drug functions (SS + DF)	670	179
17 molecular descriptors + drug functions (MD + DF)	670	29
SMILES strings + 17 molecular descriptors + drug functions (SS + MD + DF)	670	196

### 4.2 Multilabel synthetic minority over-sampling technique (MLSMOTE)

In the present work, MLSMOTE has been applied to address the issue of data under representation. It augments the data by generating synthetic data samples. The reason for selecting MLSMOTE is that it generates the data samples considering the multi-label nature of the given data. Initially it selects the minority labels. A minority label is that label corresponding to which on-an-average data samples are comparatively lower than other labels. After that all the samples corresponding to the minority labels are marked as minority samples. A data sample is selected randomly from the set of minority samples and then its *k*-nearest neighbors are selected as reference neighborhood. The attributes and labels of the new data sample are generated using interpolation method and majority voting technique respectively considering randomly selected minority samples and its reference neighborhood. In the present work, the value of *k* has been taken 7.

### 4.3 Multi-label deep neural network (MLDNN)

A deep neural network is a neural network which comprises multiple hidden layers and an output layer. An MLDNN is a DNN which consist of same number of nodes in output layer as the number of labels in the given dataset. In the present work an MLDNN model has been trained for ADR prediction having 2 hidden layers where first hidden layer have 1024 hidden nodes and second hidden layer have 2048 hidden nodes, and ReLU activation function in both hidden layers. The model is trained with a data batch size of 64 for 15 numbers of epochs considering binary cross entropy as loss function and Adam optimizer. Further, the dropout is added between the hidden layers to reduce the over-fitting of the DNN model with 0.4 dropout rate. As the number of labels in the given datasets are 6123 so output layer is designed with 6123 nodes where each node uses sigmoid function as activation function. The above mentioned values of parameters have been set by performing experimental analysis.

## 5 Experimental results and analysis

This sections presents the analysis of performance of the proposed methodology on SS, MD, DF, SS + MD, SS + DF, MD + DF and SS + MD + DF datasets in terms of precision, Recall, F1-score, ROC-AUC and HL as shown in Table 2. For this purpose, a *k*-cross validation with *k* = 5 has been implemented as training–testing strategy. As it helps in avoiding the over fitting.

**Table 2:** Performance of the proposed methodology.

Drug properties	Precision (%)	Recall (%)	F1-score	ROC-AUC	HL
SS	96.96 ± 0.0013	99.60 ± 0.0015	98.26 ± 0.0008	99.90 ± 0.0019	0.16 ± 0.0008
MD	77.34 ± 0.0063	47.96 ± 0174	59.22 ± 0.0122	97.32 ± 0.0019	3.97 ± 0.0006
DF	97.96 ± 0.0016	99.54 ± 0.0008	98.73 ± 0.0008	99.90 ± 0.0001	0.13 ± 0.0001
SS + MD	82.46 ± 0.0060	57.28 ± 0.0192	67.58 ± 0.0148	98.52 ± 0.0013	2.85 ± 0.0060
**SS + DF**	**98.64 ± 0.0016**	**99.92 ± 0.0004**	**99.20 ± 0.0012**	**99.99 ± 0.0012**	**0.09 ± 0.0001**
MD + DF	80.42 ± 0138	54.34 ± 0300	64.80 ± 0.0208	98.08 ± 0.0016	3.55 ± 0.0013
SS + MD + DF	95.91 ± 0.0063	93.26 ± 0.0192	94.54 ± 0.0107	99.90 ± 0.0001	0.64 ± 0.0012

It can be observed from Table 2 that the performance of the proposed methodology is comparable on DF and SS datasets and poor on MD dataset in comparison to them. The reason for this is that the discriminating capability of molecular drug descriptors is comparatively low because of their drug alike properties because of which DNN gives high misclassification error in case molecular descriptors as opposed to SMILES Strings and Drug Functions.

**Figure 4:**
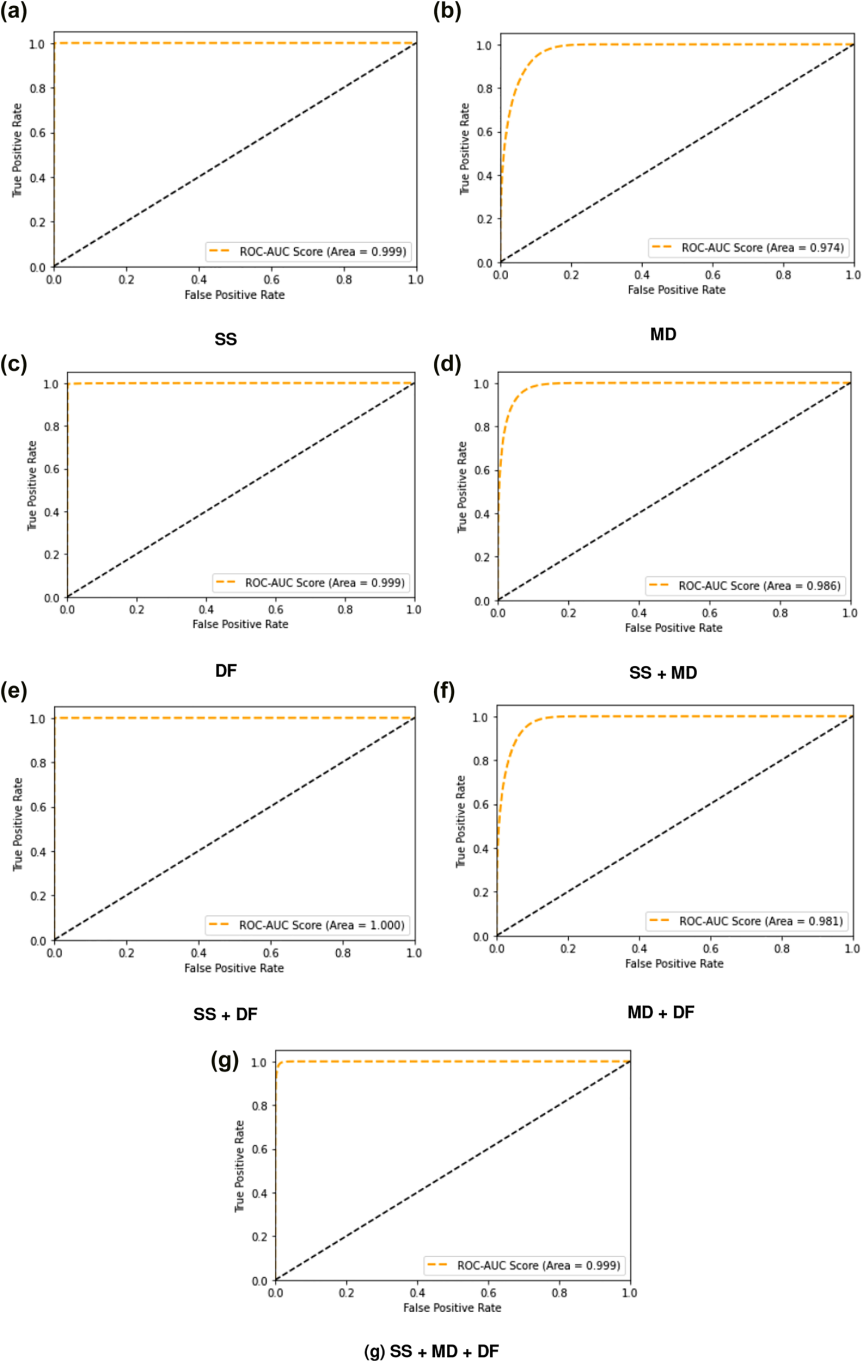
ROC-AUC for different datasets.

Further, on integrating SS with DF, the performance is enhanced whereas on integrating SS and DF with MD, the performance degraded. Even on integrating MD with SS + DF, the performance degraded. The reason for this degradation is that features of MD data are not co-relating well with features of SS and DF data and are acting as random noise whereas features of SS and DF data are co-relating properly with each other’s features and hence have more capability to differentiate between samples corresponding to different labels. Overall, the proposed methodology has achieved highest precision, recall, F1-score, ROC-AUC and lowest HL on SS + DF dataset which shows that the ADR prediction capability of SS and DF data jointly is better than SS, MD and DF individually and then integrated dataset viz. SS + MD, MD + DF and SS + MD + DF. The same observation can be made from the detailed view of ROC-AUC for the fifth validation iteration of the proposed methodology for all seven datasets as shown in Figure 4.

In conclusion, the seven datasets can be ranked as follows in terms of their prediction capability towards ADRs:

*MD* < (*MD* + *DF*) < (*MD* + *SS*) < *SS* < *DF* < (*SS* + *MD* + *DF*) < (*SS* + *DF*)

Further, the effectiveness of MLSMOTE in handling data under representation has been analyzed by comparing performance of the MLDNN with MLSMOTE and without MLSMOTE in terms of precision, Recall, F1-score, ROC-AUC and HL as given in Table 3. It can be observed from Table 3 that there is rise of 38.01% in precision, 78.28% in recall, 70.88 in F1-score and 11.08 in ROC-AUC and fall of 1.31 in HL. Based on the above discussion, it can be said that the data under representation is an acute challenge in ADR prediction. It must be handled before training and DL model. MLSMOTE has performed fairly well in addressing this challenge.

**Table 3:** Performance comparison of MLDNN with and without MLSMOTE.

	Precision (%)	Recall (%)	F1-score	ROC-AUC	HL
MLDNN without MLSMOTE	57.90 ± 0.0310	14.98 ± 0.0240	23.66 ± 0.0301	88.82 ± 0.0137	1.95 ± 0.0006
MLDNN with MLSMOTE	95.91 ± 0.0063	93.26 ± 0.0192	94.54 ± 0.0107	99.90 ± 0.0001	0.64 ± 0.0012

## 6 Conclusions

In the present work, an MLSMOTE and DNN based methodology has been presented for analysing the ADR prediction capability of chemical drug properties viz. SMILE Strings and 17 molecule descriptors and drug functions individually and in integrated manner. MLSMOTE technique has been deployed for handling the issue of data under representation and DNN models have been trained for ADR predictions. The proposed methodology has been validated on seven datasets namely SS, MD, DF, SS + MD, SS + DF, MD + DF and SS + MD + DF. Based on the validation results, the datasets are ranked as *MD* < (*MD* + *DF*) < (*MD* + *SS*) < *SS* < *DF* < (*SS* + *MD* + *DF*) < (*SS* + *DF*) with regard to their ADR prediction capability. Further, the validation results signify the effectiveness of MLSMOTE technique in handling data under representation for multi-label dataset.
